# Partially coherent light propagation through a kinoform lens

**DOI:** 10.1107/S1600577523000875

**Published:** 2023-03-22

**Authors:** Weihong Sun, Yong Wang, Xiangyu Meng, Junchao Ren, Jiefeng Cao, Junqin Li, Renzhong Tai

**Affiliations:** a Shanghai Institute of Applied Physics, Chinese Academy of Sciences, Jialuo Road 2019, Jiading District, Shanghai 201800, People’s Republic of China; b University of Chinese Academy of Sciences, Yuquan Road 19, Shijingshan District, Beijing 100049, People’s Republic of China; c Shanghai Advanced Research Institute, Chinese Academy of Sciences, Zhangheng Road 239, Pudong District, Shanghai 201204, People’s Republic of China; University of Tokyo, Japan

**Keywords:** synchrotron radiation beamline, kinoform lens, mutual optical intensity, partially coherent light

## Abstract

Combining wave optics propagation and geometric ray tracing, the mutual optical intensity (MOI) model is extended to quantitatively simulate the propagation of partially coherent light through a kinoform lens at high speed. The MOI model can provide both a high accuracy and a high efficiency simulation. The intensity and coherence degree distributions at the focal plane are calculated using the MOI model. It is beneficial to improve the focusing capability of the kinoform lens by reducing the coherence or increasing the number of lens steps. In addition, increasing the number of steps is also beneficial to increase the photon flux and reduce the depth of focus.

## Abstract 2

The mutual optical intensity model is extended to calculate the propagation of partially coherent X-rays through a kinoform lens.

## 1. Introduction

With the development of free-electron lasers (Emma *et al.*, 2010 ▸; Ishikawa *et al.*, 2012 ▸; Hettel, 2014 ▸) and diffraction-limited storage rings (Allaria *et al.*, 2012 ▸; Eriksson *et al.*, 2014 ▸; de Jonge *et al.*, 2014 ▸), the coherence of synchrotron radiation X-rays has greatly improved (Qi *et al.*, 2014 ▸), which can advance the performance of coherent diffraction imaging (Liang *et al.*, 2015 ▸), X-ray photon correlation spectroscopy (Stephenson *et al.*, 2009 ▸), coherent X-ray scatter imaging (Ice *et al.*, 2011 ▸) and so on. These techniques not only rely on advanced X-ray sources but also on high efficiency focusing optical elements. Refractive focusing elements can perform submicrometre focusing in the hard X-ray energy range while keeping the characteristics of high theoretical working energy and flexibility (Snigirev & Snigireva, 2008 ▸). However, due to the strong absorption of X-rays by the edges of conventional X-ray refractive focusing elements such as compound refractive lenses, the photon flux and focusing capability cannot be improved by increasing the geometric aperture. Based on the plano-concave lens, the kinoform lens is realised by removal of passive material where the path length is an integer multiple of the X-ray wavelength, which effectively increases the geometric aperture (Aristov *et al.*, 2000 ▸). At present many models can be used to optimize the design of kinoform lenses, but with some disadvantages. For example, the thin-lens approximation method (Buralli *et al.*, 1989 ▸) is a simple model with low calculation accuracy. The Takagi–Taupin description (TTD) (Yan *et al.*, 2007 ▸) of X-ray dynamical diffraction theory is a rigorously accurate method that is only suitable for the analysis of short kinoform lenses (Yan, 2010 ▸). The beam propagation method (Van Roey *et al.*, 1981 ▸) is based on dividing the object into slices, and angular spectral theory has high calculation accuracy and low calculation efficiency (Yan, 2010 ▸). However, the existing model is only suitable for fully coherent light, and cannot simulate the propagation of partially coherent light through a kinoform lens.

Based on statistical optics, we have established the MOI model (Meng *et al.*, 2015 ▸; Ren *et al.*, 2019 ▸) that simulates the propagation of partially coherent light through synchrotron radiation beamlines. The model can provide the intensity, coherence and phase information of the partially coherent light at a specified position on the beamline. In this work, combining wave optics propagation and geometric ray tracing, the MOI model is extended to simulate the propagation of partially coherent light through a kinoform lens. The effects of the coherence and the number of kinoform lens steps on the focusing capability are quantitatively analyzed.

## 2. Model description

### 2.1. Establishing the MOI model for a kinoform lens

**Figure 1:**
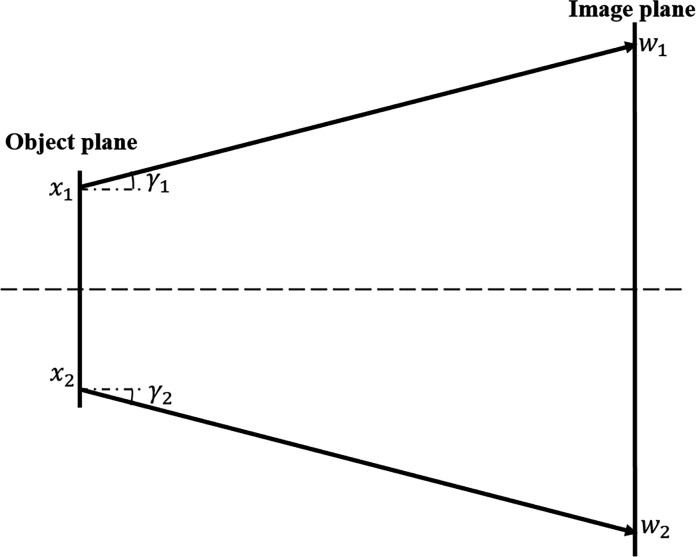
Schematic diagram of mutual optical intensity propagation through free space.

Since the long kinoform lens is widely applied in hard X-ray submicrometre focusing, we take the long kinoform lens as an example to introduce the MOI model. The refractive index of a plano-concave lens is given by *n* = 1 − δ + *i*β, where δ is the refractive coefficient of the medium and β is the attenuation coefficient of the medium. X-rays can be focused by the plano-concave lens. Based on the plano-concave lens, the kinoform lens is realised by removal of passive material where the path length is an integer multiple of the X-ray wavelength, which not only maintains the focusing characteristics but also improves the X-ray transmittance. The step length, *l* = *m*λ/δ, generates an *m*-integer-2π phase difference between X-ray propagation inside and outside the step, where λ is the wavelength and *m* is a positive integer. The curved surface of the long kinoform lens is elliptical and can be expressed as (Evans-Lutterodt *et al.*, 2003 ▸)

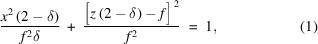

where *x* is the transverse coordinate of the lens, *z* is the coordinate along the optical axis, and *f* is the focal length. The MOI model uses the mutual optical intensity to describe the partially coherent light. The X-ray transmission from the upstream source to the incident surface of the kinoform lens and from the exit surface to the focal plane are free-space propagation. The mutual optical intensity propagation through free space can be written as (Born *et al.*, 1959 ▸; Goodman, 2015 ▸)

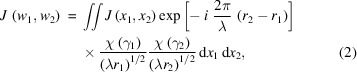

where λ is the wavelength, *x*_1_ and *x*_2_ are any two points on the object plane, *w*_1_ and *w*_2_ are any two points on the image plane, *r*_1_ and *r*_2_ are the *x*_1_-to-*w*_1_ and *x*_2_-to-*w*_2_ distances, respectively, χ(γ_1_) and (γ_2_) are the inclination factors for the inclination angle γ_1_ and γ_2_, respectively, and *J*(*x*_1_, *x*_2_) and *J*(*w*_1_, *w*_2_) are the mutual optical intensity at the object and the image planes, respectively. A schematic diagram of the mutual optical intensity propagation through free space is shown in Fig. 1 ▸.

The mutual optical intensity propagation through free space based on formula (2) is numerically calculated. The MOI model calculation procedure is built as follows. Firstly, the source plane is divided into many small elements. Each element is small enough to be considered to have full coherence and constant intensity. Secondly, the propagation of mutual optical intensity in each element is carried out using the Fresnel approximation or the Fraunhofer approximation (Born *et al.*, 1959 ▸). Finally, the mutual optical intensity at the image plane can be obtained by summing the contributions of all elements. The mutual optical intensity propagation through free space can be expressed as (Ren *et al.*, 2019 ▸)

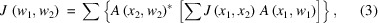

where *A*(*x*_1_, *w*_1_) is the integral of *x*_1_ on the object plane as follows,

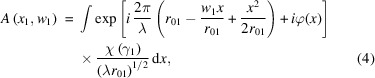

where *r*_01_ is the distance between the center points of the two elements *x*_1_ and *w*_1_, and φ(*x*) is the phase distribution within each element.

**Figure 2:**
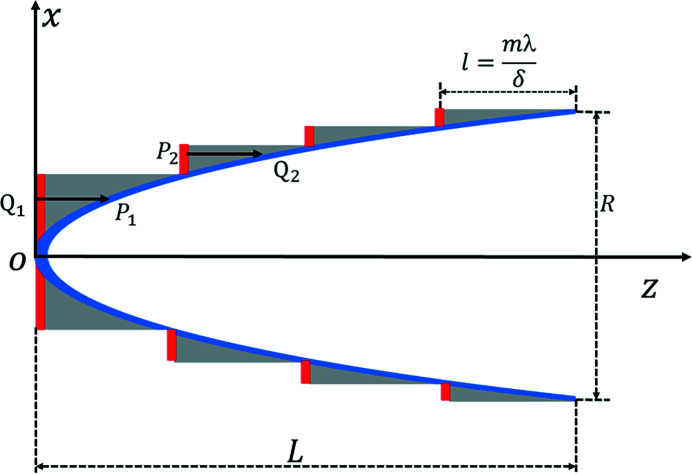
Schematic diagram of a long kinoform lens. The *x* and *z* axes denote the transversal and longitudinal directions, *L* denotes the total length of the lens, *R* denotes the aperture of the lens, *l* denotes the length of each step, and *P* and *Q* denote points at the incident and exit surfaces.

The incident surface (red lines) is defined as the upstream surface of the kinoform lens, as shown in Fig. 2 ▸. The exit surface (blue curve) is defined as the back surface of the kinoform lens. The incident and exit surfaces are described by the *P* and *Q* planes. The geometrical tracing method is used to analyze the wavefront propagation through the incident surface to the exit surface. The wavefront at the incident surface is divided into many small surface elements. The rays travel along the direction defined by the phase gradient within each element at the incident plane. Following Fermat’s principle (Born & Wolf, 1999 ▸), the rays enter the kinoform lens medium, transmit through the medium, and hit the exit plane. The path length of the ray traveling inside the kinoform lens is used to describe the phase change. Assume that a certain ray enters the medium from point *P* at the incident surface and leaves the medium from point *Q* at the exit surface. *J*(*P*_1_, *P*_2_) is the mutual intensity of the incident surface, and *J*(*Q*_1_, *Q*_2_) is the mutual intensity of the exit surface, which can be expressed as

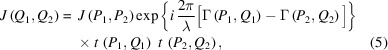




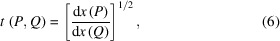




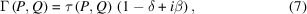

where *t*(*P*, *Q*) is the complex amplitude transmission function; *x*(*P*) and *x*(*Q*) are the coordinates of *P* and *Q*, respectively; τ(*P*, *Q*) is the distance from point *P* to point *Q*; 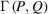 is the path length from point *P* to point *Q*, which describes the phase change and attenuation of the wavefront by the medium. Since the wavefront at the incident surface of the kinoform lens is not a plane wave, some rays may escape from the step edge of the kinoform lens. For simplicity, the escaped rays are ignored in the subsequent propagation. The total lost flux in the whole kinoform is less than 0.1%. Therefore, the MOI model still has high accuracy. The real component 1−δ of the refractive index for the silicon medium is very close to 1. The phase shift 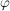 = 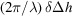 is very small from the figure error or roughness Δ*h*. Unlike reflective optical elements such as Kirk­patrick–Baez mirrors, the figure error and roughness on the kinoform lens have negligible effect on damage of the wavefront. For other refractive optical elements such as compound refractive lenses, similar conclusions have also been given (Pantell *et al.*, 2001 ▸). In this paper, the effect from optics-related errors is not considered.

**Figure 3:**
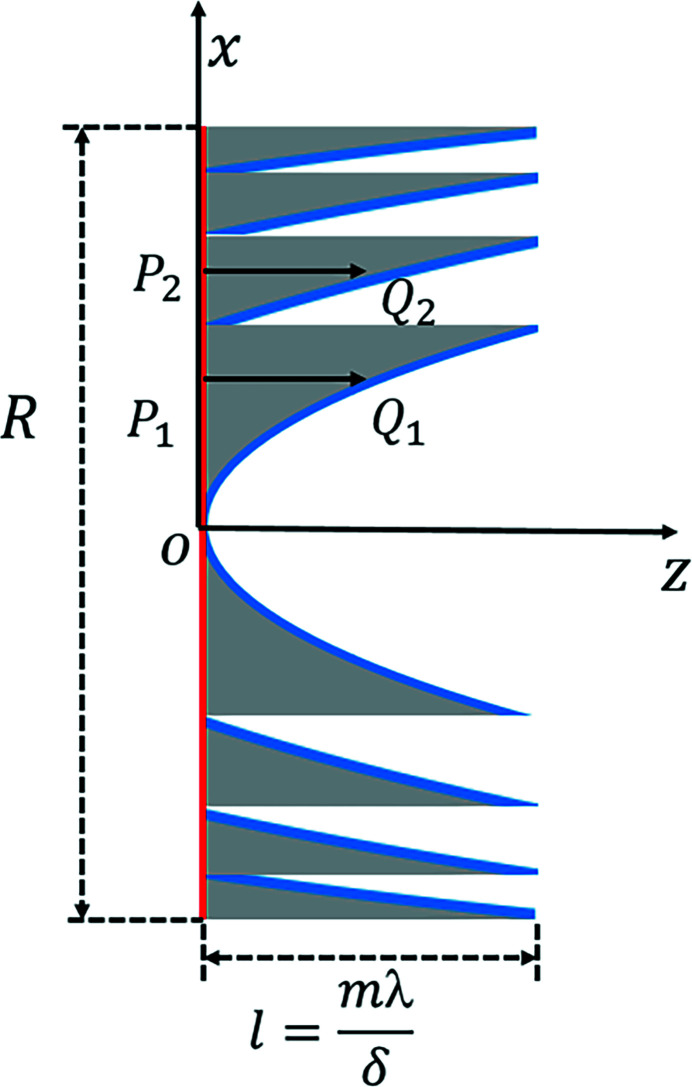
Schematic diagram of a short kinoform lens. The *x* and *z* axes denote the transversal and longitudinal directions, *R* denotes the aperture of the lens, *l* denotes the total length of the lens, *P* and *Q* denote points at the incident and exit surfaces.

Unlike the long kinoform lens, the steps for the short kinoform lens are designed for one plane, as shown in Fig. 3 ▸. The thickness of the short kinoform lens is *l* = *m*λ/δ. The left plane (red line) is the incident plane, and the right curved surface (blue curve) is the exit surface. The exit surface function of different steps for the short kinoform lens is hyperbolic, while the surface function for the long kinoform is elliptical. The formula for the short kinoform lens can be expressed as follows (Cao *et al.*, 2016 ▸),

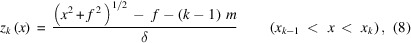

where *k* is the step number. The wavefront propagation through the short kinoform lens is also defined by formula (5).

The propagation of mutual optical intensity through the lens can be performed in three steps: firstly, free-space propagation from the source plane to the incident plane using formula (3); secondly, propagation from incident surface to the exit surface using formula (5); and, finally, propagation from the exit surface to the focal plane using formula (3).

### 2.2. Gaussian Schell model

The mutual optical intensity at the source plane can be described by the Gaussian Schell model (GSM) (Gori *et al.*, 2001 ▸; Starikov & Wolf, 1982 ▸) and is given as

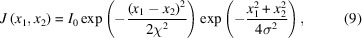

where *J*(*x*_1_, *x*_2_) is the mutual optical intensity between two points *x*_1_ and *x*_2_ at the source plane, χ represents the coherence length, σ represents the root-mean-square (r.m.s.) beam size and *I*_0_ represents the optical intensity at the central point. Note that the GSM is not necessary for the MOI code, and any mutual optical intensity distribution can be used for the calculation. The global degree of coherence of the source is described by the following formula (Vartanyants & Singer, 2010 ▸),

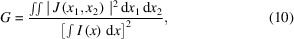

where *I*(*x*) denotes the intensity distribution at the source plane.

## 3. Simulation results

### 3.1. Simulation of partially coherent light propagation through a long kinoform lens

**Figure 4:**
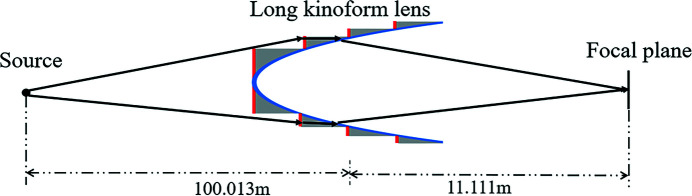
Optical setup for the long kinoform lens.

The MOI model is used to simulate the partially coherent light propagation through a long kinoform lens. The optical setup is shown in Fig. 4 ▸. The mutual optical intensity at the source plane is obtained by using formula (9). The GSM source energy is 10 keV with beam r.m.s. size of σ = 10 µm and coherence length of χ = 11.5 µm, and the global degree of coherence *G* is 0.50. The source-to-kinoform and kinoform-to-image distances are 100.013 m and 11.111 m, respectively. The aperture of the kinoform lens *R* is 3.18 mm. The total length of the lens *L* is 25.96 mm with each step length 203 µm; the step number of the lens is 128, and the material of the lens is silicon. When the energy is 10 keV, δ and β are 1.2398 × 10^−6^ and 7.3841 × 10^−8^, respectively. The focal length of the lens is 10 m.

**Figure 5:**
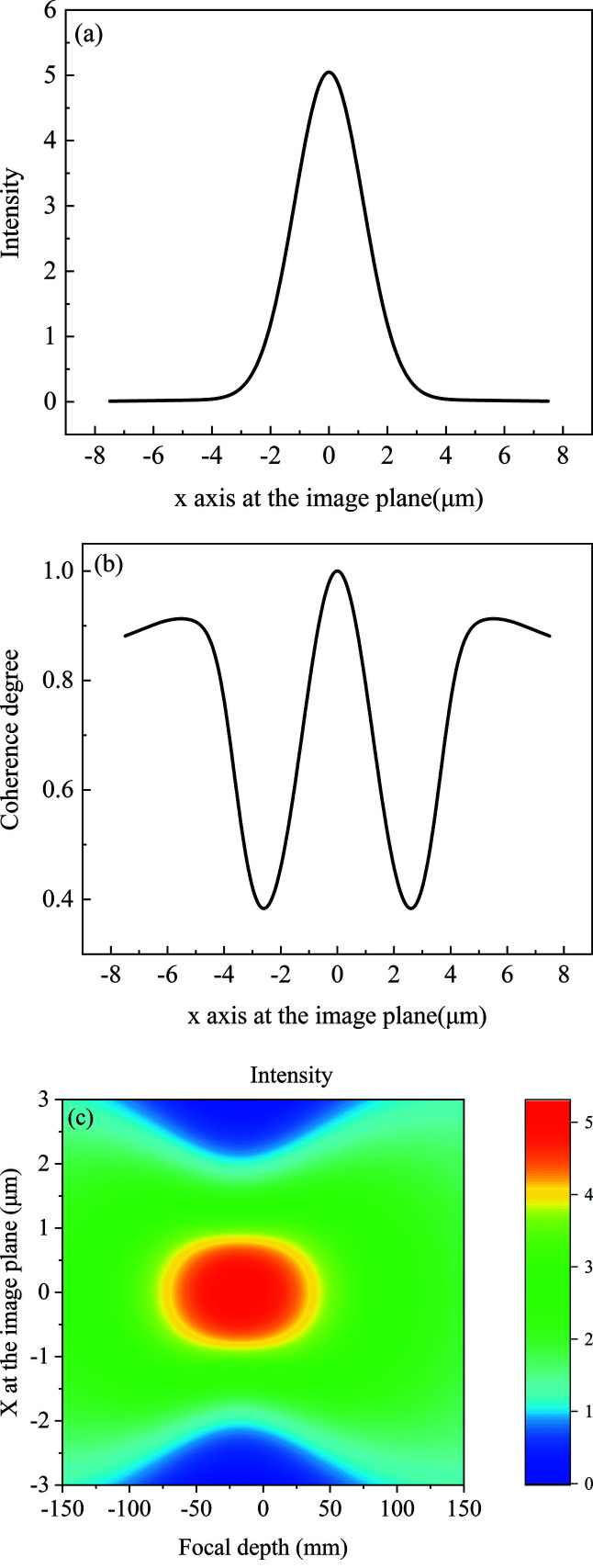
Partially coherent light propagation through the long kinoform lens. (*a*) Intensity distribution at the focal plane, (*b*) coherence degree distribution at the focal plane, (*c*) intensity distribution along the focus depth. The coherence degree denotes the correlation between any point and the center point.

The incident surface is divided into 5000 elements with each element of size 0.64 µm. The intensity and coherence degree distribution at the focal plane are shown in Figs. 5 ▸(*a*) and 5 ▸(*b*). The focus spot r.m.s. size is 1.17 µm, calculated using the MOI model, which is very close to the spot size of 1.11 µm estimated by the geometrical optics. The reason for the slight difference in the spot size is that the geometrical optics cannot simulate the diffraction effect of the kinoform lens structure on the focus spot. The X-ray transmission varies from 100% to 31% due to the stepped structure of the kinoform lens. The diffraction effect from the stepped structure results in a slight enhancement of the global degree of coherence to 0.55 at the focal plane. The apparent oscillation peaks on the edges of the coherence degree profile are caused by the diffraction effect of the kinoform lens stepped structure. The MOI model has high calculation efficiency. It can be used to calculate the intensity distribution along the optical axis, as shown in Fig. 5 ▸(*c*). The focus depth is approximately 240 mm. The minimal focus spot located at −18 mm is due to the flat plane wave of the source and the diffraction effect of the kinoform lens stepped structure. In our previous paper (Sun *et al.*, 2022 ▸), the ladder wavefront (LWF) model can be seen as a special MOI model under the full coherence condition. The spot intensity at the focal plane calculated from the LWF model is in good agreement with that calculated from the beam propagation method model. Therefore, the MOI model has high accuracy.

**Figure 6:**
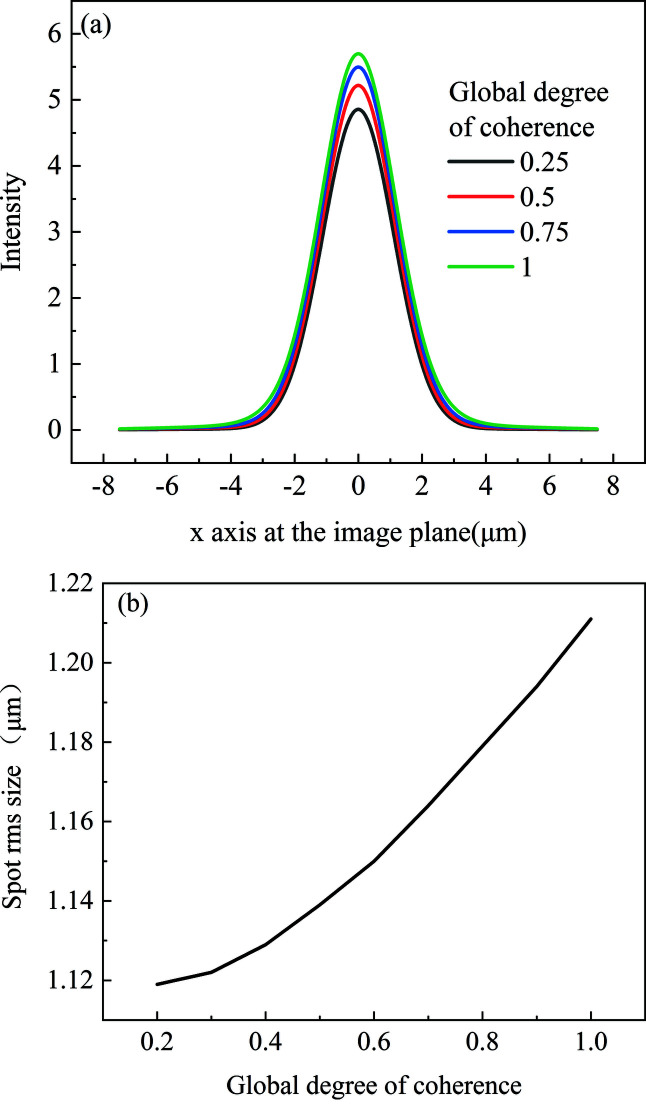
(*a*) Intensity distribution at the focal plane with different global degrees of coherence. (*b*) Spot size at the focal plane as a function of global degree of coherence.

The source spot size is σ = 10 µm. The coherence length is chosen to be 5.2 µm, 11.5 µm, 22.7 µm and ∞, with corresponding global degree of coherence of 0.25, 0.5, 0.75 and 1. The intensity distribution at the focal plane corresponding to the minimum focus spot is shown in Fig. 6 ▸(*a*). When the global degree of coherence is 0.25, the spot r.m.s. size at the focal plane is 1.12 µm. With increasing coherence length, the spot size at the focal plane increases gradually. When the global degree of coherence is 1, the spot r.m.s. size at the focal plane is 1.21 µm. The source divergence angle is inversely proportional to the global degree of coherence (Vartanyants & Singer, 2010 ▸). To make the full beam accepted by the kinoform lens with aperture of 3.18 mm, the minimum global degree of coherence should be greater than 0.2. The spot size as a function of the global degree of coherence is shown in Fig. 6 ▸(*b*). The greater the global degree of coherence, the stronger the diffraction effect from the stepped structure. Therefore, the spot size at the focal plane gradually rises with increasing global degree of coherence.

The kinoform lens is composed of many steps. The number of steps is related to the thickness of the steps. A fewer number of steps reduces the light transmission of the lens, while a higher number of steps greatly increases the difficulty of kinoform lens fabrication. At present the shortest processing length of kinoform lens steps is 0.4 µm (Xu *et al.*, 2018 ▸); therefore, the effect of the number of steps on the focusing capability of the long kinoform lens needs to be analyzed.

**Figure 7:**
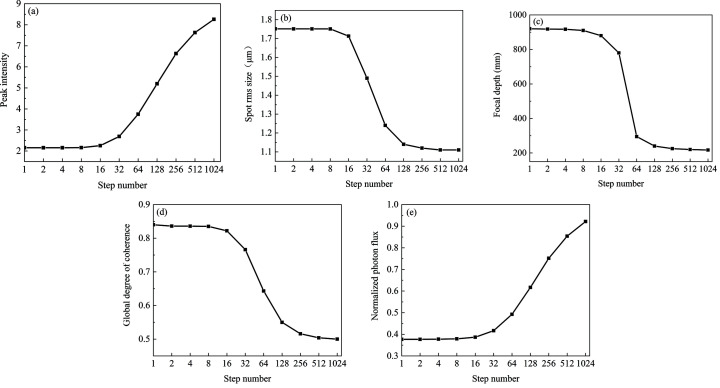
Focusing performance of a long kinoform lens as a function of the number of steps. (*a*) Peak intensity, (*b*) spot r.m.s. size, (*c*) focal depth, (*d*) global degree of coherence and (*e*) normalized photon flux with the various numbers of steps.

The GSM source parameters are σ = 10 µm and χ = 11.5 µm, and the global degree of coherence is *G* = 0.50. The source energy is 10 keV. The kinoform lens has the same specific parameters as in the previous paragraph. We analyze the effect of the number of steps *k* varying from 1 to 1024 on the focusing capability. Peak intensity, spot r.m.s. size, focal depth, global degree of coherence and normalized photon flux at the position of the minimum focus spot are shown in Figs. 7 ▸(*a*)–7 ▸(*e*). The normalized photon flux is the ratio between the photon flux at the focal plane and the source plane. As the number of steps increases and the length of the steps decreases, the light transmission difference for various positions of each step gradually decreases. As the number of steps *k* varies from 1 to 1024, the minimum light transmittance of the kinoform lens stepped structure increases from 0% to 86%. Therefore, the diffraction effect of the steps decreases with increasing step number, which is beneficial to increasing the focusing capability and reducing the focus depth. Figs. 7 ▸(*a*), 7 ▸(*b*) and 7 ▸(*e*) show that the peak intensity increases, the spot size decreases and the normalized photon flux increases with increasing step number. When the step number is 128, the light transmission changes with the position of one step from 31% to 100%. When the step number is greater than 128, the spot size r.m.s. of 1.14 µm tends to be stable. At the same time, the focus depth as a function of step number is shown in Fig. 7 ▸(*c*). When the number of steps is 128, the focal depth is 240 mm, which tends to be stable. Since the diffraction effect of the steps decreases with increasing step number, the global degree of coherence decreases with increasing step number as shown in Fig. 7 ▸(*d*). When the number of steps is 128, the global degree of coherence at the focal plane is 0.55, which is close to stable. Increasing the number of steps is beneficial to improve the focusing capability, increase the photon flux and reduce the focal depth, but it also increases the manufacturing difficulty. Selecting the appropriate number of steps is very important for the design of a kinoform lens. In this case, our analysis shows that 128 steps are good enough to achieve a high-focusing performance. All MOI simulations were performed on a laptop with i5-9300h CPU and 16 GB RAM. The element numbers of the wavefront for different planes are chosen to be 5000, which is large enough to achieve high accuracy calculation. The total calculation time from the source to focal planes is 4 s. Therefore, the MOI model can provide both a high accuracy and a high efficiency simulation.

### 3.2. Simulation of partially coherent light propagation through a short kinoform lens

**Figure 8:**
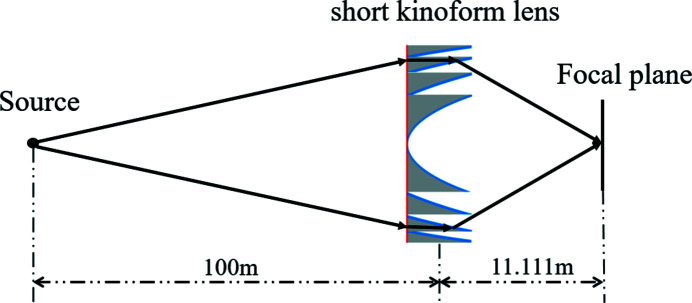
Optical setup for the short kinoform lens.

**Figure 9:**
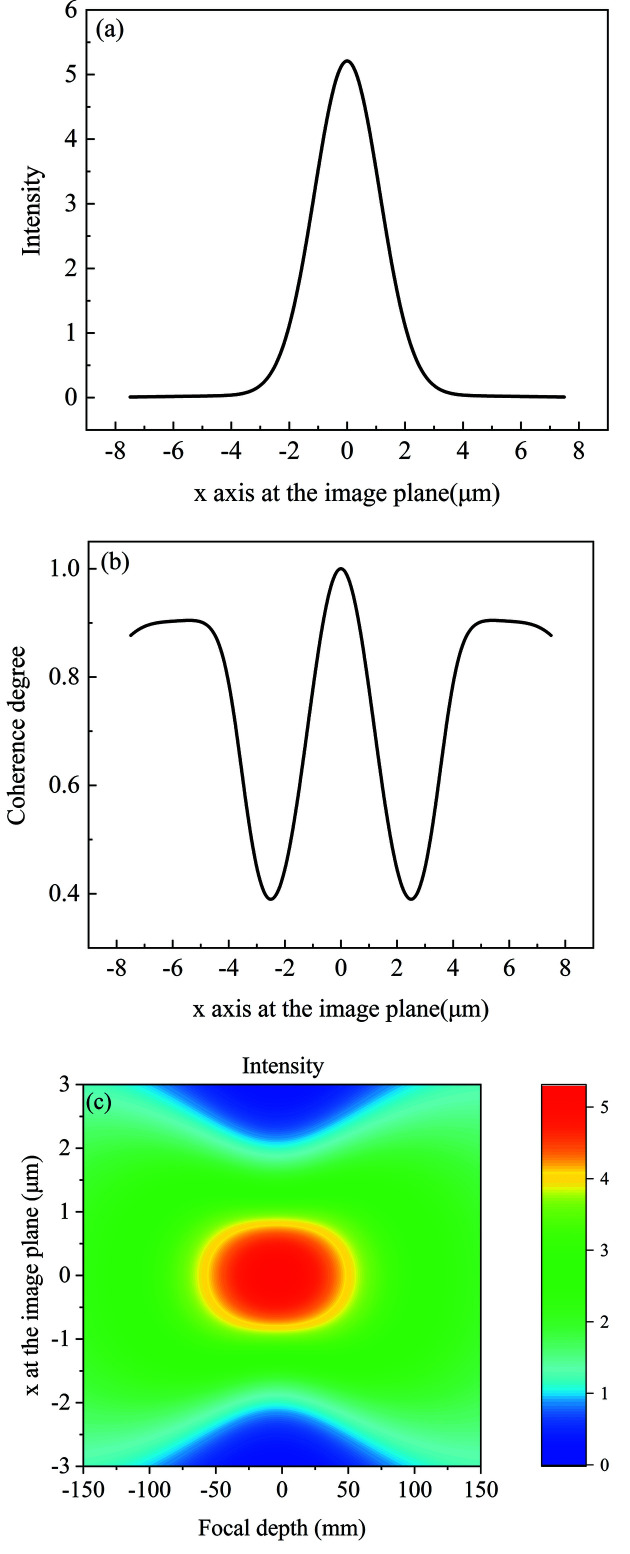
Partially coherent light propagation through the short kinoform lens. (*a*) Intensity distribution at the focal plane, (*b*) coherence degree distribution at the focal plane and (*c*) intensity distribution along the focus depth.

The MOI model is used to simulate the propagation of partially coherent light through a short kinoform lens. The parameters of the source are the same as those given in Section 3.1. The optical setup is shown in Fig. 8 ▸. The source-to-lens and lens-to-focal distances are 100 m and 11.111 m, respectively. The aperture of the kinoform lens, *R*, is 3.19 mm, the total length of the lens, *l*, is 203 µm and the step number of the lens is 128. The focal length of the lens is 10 m. The intensity and coherence degree distribution at the focal plane are shown in Figs. 9 ▸(*a*) and 9 ▸(*b*). The focus spot r.m.s. size is 1.14 µm which is close to the spot size of 1.11 µm estimated by the geometrical optics. The spot sizes are slightly different for the long and short kinoform lens. This is due to the difference between the long and short kinoform lens. The short kinoform lens is more like an ideal lens than the long one because its length along the beam is shorter than that of the long one. Therefore, the actual focus point of the long kinoform lens is farther away from the theoretical focus than the short one. We calculated the intensity profile at the theoretical position, so the focal spot size of the short kinoform lens is smaller than that of the long one. Like the long kinoform lens, the stepped structure of the kinoform lens causes a diffraction effect. This diffraction effect results in a slight enhancement of the global degree of coherence of 0.55 at the focal plane. The intensity distribution along the focus depth is shown in Fig. 9 ▸(*c*). The focus depth is 240 mm. There is a 3.6 mm position difference between the simulated and geometrical focal planes.

**Figure 10:**
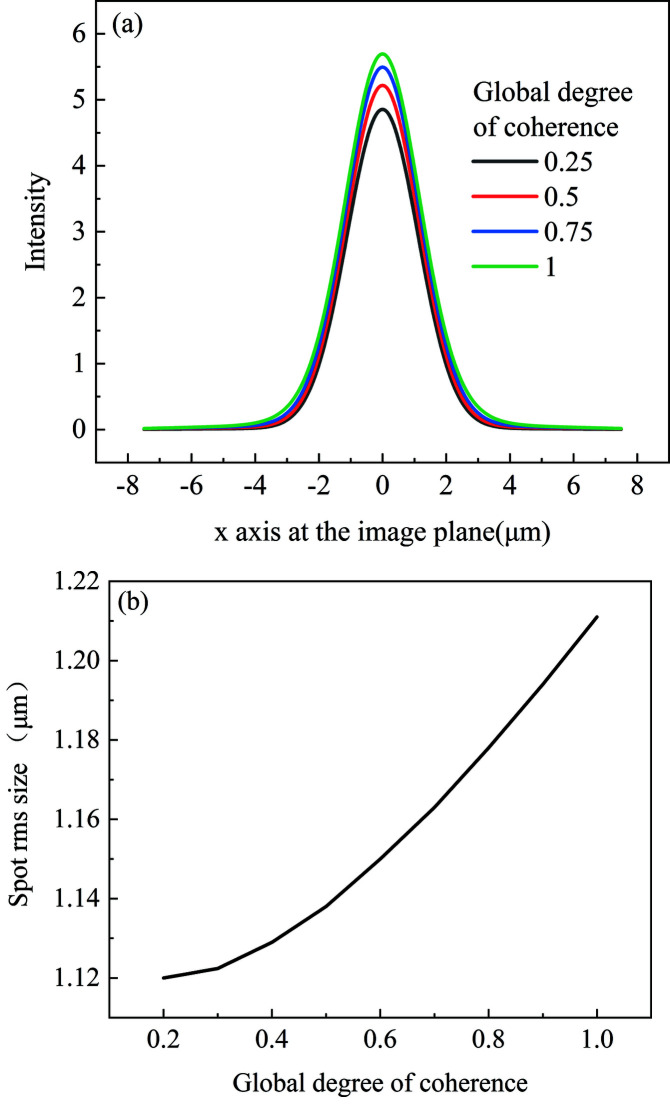
(*a*) Intensity distribution at the focal plane with different global degrees of coherence. (*b*) Focus spot size for various global degrees of coherence.

The source spot size is σ = 10 µm. The coherence length is chosen to be 5.2 µm, 11.5 µm, 22.7 µm and ∞, with corresponding global degree of coherence of 0.25, 0.5, 0.75 and 1. The intensity distribution at the plane for the minimum focus spot is shown in Fig. 10 ▸(*a*). The focus spot size with various global degrees of coherence is shown in Fig. 10 ▸(*b*). Like the long kinoform lens, the focus spot size gradually increases as the global degree of coherence increases.

**Figure 11:**
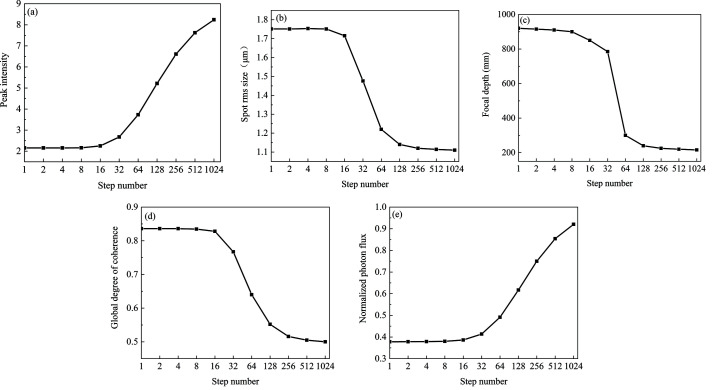
Focusing performance of the short kinoform lens with various numbers of steps. (*a*) Peak intensity, (*b*) spot r.m.s. size, (*c*) focal depth, (*d*) global degree of coherence and (*e*) normalized photon flux as a function of the number of steps at the focal plane.

We simulate the effect of the number of steps on the focusing capability of the short kinoform lens. The parameters of the source are the same as those given in Section 3.1. The aperture of the lens is *R* = 3.19 mm, the focal length is *f* = 10 m and the total length of the lens is *L* = 203 µm. Peak intensity, spot r.m.s. size, focal depth, global degree of coherence and normalized photon flux at the focal plane with various step numbers *k* are shown in Figs. 11 ▸(*a*)–11(*e*). Like the long kinoform lens, increasing the number of steps is beneficial to improve the focusing capability, increase the photon flux and reduce the focal depth. When the number of steps is larger than 128, the spot r.m.s. size, focal depth and global degree of coherence at the focal plane tend to be stable. Therefore, 128 steps are sufficient to achieve high focusing performance.

## 4. Conclusion

In this paper, the MOI model is developed to simulate the propagation of partially coherent light through the long and short kinoform lenses with high speed. The simulation can be performed within several seconds which is practically useful for the optical design of kinoform lenses. Reducing the coherence is beneficial to improve the focusing capability of the kinoform lens. Increasing the number of steps is beneficial to improve the focusing capability of the kinoform lens, increase the photon flux and reduce the focus depth. The MOI model is a useful tool for the design of kinoform lenses in synchrotron radiation beamlines.
